# The Effect of Perioperative Thermoregulation on Cancer Surgeries: A Narrative Review

**DOI:** 10.7759/cureus.97870

**Published:** 2025-11-26

**Authors:** Chetla Rakesh, Anagani Hrushikesh, C.V.K.K Chaitanya, Vusirikayala Naga Sireesha

**Affiliations:** 1 Department of Emergency Medicine, Amrita Institute of Medical Sciences and Research, Kochi, IND; 2 Department of Anesthesia, Centurion University of Technology and Management, Vizianagaram, IND; 3 Department of Public Health, Amrita Institute of Medical Sciences and Research, Kochi, IND

**Keywords:** "anaesthesia", hypothermia, intraoperative warming techniques, normothermia, oncology surgery, perioperative thermoregulation, postoperative care, preoperative warming, surgical site infections (ssi), temperature monitoring

## Abstract

Owing to the vulnerability of adverse effects of hypothermia, particularly among immunocompromised cancer patients, perioperative thermoregulation is critical. This review presents a comprehensive examination of current thermoregulation practices, including preoperative, intraoperative, and postoperative phases.

We conducted literature search for relevant articles using PubMed, MEDLINE, EMBASE, Cochrane Library, Scopus, Google Scholar and Web of Science, published till June 2024 using the keywords, "Perioperative thermoregulation," "cancer surgery," "hypothermia," "normothermia," "surgical site infections," "intraoperative warming techniques," "preoperative warming," "postoperative care," and "patient outcomes".

Our review highlights the adverse impact of hypothermia among cancer patients, such as increased surgical complications, impaired immune response, and prolonged recovery times. This review also shines light on the efficacy of various perioperative warming techniques, such as forced-air warming systems and fluid warming methods. Additionally, this review article addresses the challenges associated with perioperative thermoregulation in cancer surgeries and explores potential innovations and future directions in this field.

## Introduction and background

Maintaining normal body temperature during surgical procedures is a critical aspect of perioperative care. Normothermia, defined as the maintenance of core body temperature within the normal range (36.5°C to 37.5°C), is essential for various physiological processes [1]. A significant concern in perioperative procedures is the risk of perioperative hypothermia. Simon et al. (2021) define perioperative hypothermia as the drop in core body temperature below 36°C [2]. Perioperative hypothermia has a varied incidence rate of 4% - 70%, which can further lead to adverse events, such as an increased risk of surgical site infections (SSIs), impaired wound healing, coagulopathy, and prolonged hospitalisation, which can further exacerbate patient morbidity and increase healthcare costs [2,3].

In the context of cancer surgeries, the importance of maintaining normothermia becomes even more significant. Cancer patients often are immunocompromised with increased physiological stress, making them more vulnerable to the detrimental effects of perioperative hypothermia. Hypothermia in cancer patients can lead to several severe complications, including suppressed immune function, increased susceptibility to infections, impaired drug metabolism, and prolonged recovery. Moreover, the complexity and duration of cancer surgeries, patients' preexisting conditions, and ongoing treatments (chemotherapy and radiation therapy) can further complicate the management of perioperative temperature [4].

Given the heightened risks associated with perioperative hypothermia in cancer surgeries, it is imperative to implement effective thermoregulation strategies. These strategies in pre-, intra-, and post-operative phases aim to prevent temperature drops and maintain normothermia throughout the surgical journey. Preoperative measures include warming the patient before surgery, while intraoperative interventions involve the use of forced-air warming systems, conductive warming devices, and warmed intravenous fluids. Postoperative care focuses on continuous temperature monitoring and the application of warming techniques to ensure a stable recovery [5-7].

The relevance of thermoregulation in cancer surgeries cannot be overstated. By maintaining normothermia, healthcare providers can mitigate the risks associated with perioperative hypothermia, enhance patient safety, and improve surgical outcomes. This narrative review aims to provide a comprehensive overview of the current perioperative thermoregulation practices, highlight the unique challenges faced in cancer surgeries, and explore potential innovations to optimize patient care in this critical area.

We conducted literature search for relevant articles using PubMed, MEDLINE, EMBASE, Cochrane Library, Scopus, Google Scholar and Web of Science, published till June 2024 using the keywords, "Perioperative thermoregulation," "cancer surgery," "hypothermia," "normothermia," "surgical site infections," "intraoperative warming techniques," "preoperative warming," "postoperative care," and "patient outcomes". Relevant original research studies, meta-analyses, narrative, and systematic reviews were included while conference abstracts, editorials and non-peer-reviewed articles were excluded. No formal risk of bias assessment was performed since this was a narrative review; however, high-quality, peer-reviewed and highly cited literature only was included.

## Review

Normal thermoregulatory mechanisms

The human body maintains core body temperature typically around 36.5°C to 37.5°C, through complex thermoregulatory mechanisms governed primarily by the hypothalamus. The hypothalamus acts as the body’s thermostat, integrating thermal information from peripheral and central thermoreceptors located in the skin, spinal cord, and other tissues [2]. When the hypothalamus detects deviations from the set point temperature, it initiates various physiological responses to either dissipate or conserve heat. The various physiological responses for heat dissipation include vasodilation, sweating and increased respiration rate [2]. Various physiological responses for heat conservation include vasoconstriction, shivering, and non-shivering thermogenesis [2,3,6]. The balance between these heat production and dissipation processes ensures the maintenance of core temperature within a narrow, optimal range, essential for normal cellular and metabolic functions.

Disruptions in thermoregulation during surgery

The surgical environment poses several challenges to the body’s thermoregulatory mechanisms, thereby causing perioperative hypothermia. Key causes that contribute to the disruptions in thermoregulation during surgery include anaesthesia-induced hypothermia, exposure to a cold operating room environment, administering cold intravenous fluids, evaporative heat loss, prolonged surgical duration, physiological stress, and metabolic changes [2,8-13].

Anaesthesia significantly impairs the body’s ability to regulate temperature. Both general and regional anaesthetics inhibit the hypothalamic response, reducing vasoconstriction and shivering thresholds. This leads to increased heat dissipation and decreased heat production. The administration of neuromuscular blocking agents further enhances this effect by eliminating shivering and muscular thermogenesis, the most important mechanisms of endogenous heat production. As a result, core body temperature drops more quickly and profoundly during deep anaesthesia with muscle relaxants. Additionally, anaesthetics cause vasodilation, which promotes heat loss from the core to the periphery [2,8].

Operating rooms are typically kept at cooler temperatures to maintain a sterile environment and ensure comfort for the surgical team. However, this cool ambient temperature can lead to significant heat loss in patients, particularly through radiation and convection thereby altering core body temperature [2,9].

The infusion of cold intravenous fluids and blood products can cause a direct transfer of cold to the patient’s core, lowering core body temperature, thereby exacerbating hypothermia. Pre-warmed infusions or an infusion warming device can be used pre-procedure to prevent temperature changes [2]. A study by Andrzejowski et al. (2010) identified that administration of pre-warmed fluids prior to surgical procedures was effective in preventing post-operative hypothermia [14].

During surgery, patients are often exposed to evaporative heat loss from surgical incisions and the use of cold antiseptic solutions. This is particularly pronounced in surgeries involving large open areas, such as major abdominal or thoracic procedures. Also, surgical duration also increases the risk of cumulative heat loss. Extended exposure to the surgical environment and continuous impairment of thermoregulatory responses can result in substantial decreases in core temperature [2,10,11]. The physiological stress of surgery, including blood loss and fluid shifts, can alter metabolic rates and affect the body’s ability to generate and retain heat. Cancer patients may have compromised metabolic responses due to the disease and its treatment [15]. These thermoregulatory disturbances tend to be more marked among oncology patients, as surgeries related to cancer are often very extensive, with large exposed areas of the abdomen or thorax leading to greater heat loss. Furthermore, many cancer patients have decreased body insulation from cachexia, anaemia, or malnutrition, further augmenting anaesthesia-induced hypothermia. Chemotherapy and radiotherapy may also further reduce vascular tone and impair autonomic control to make the body even less capable of maintaining temperature stability during surgery [2,10-13].

In conclusion, understanding the normal thermoregulatory mechanisms and the factors that disrupt these processes during surgery is crucial for implementing effective perioperative thermoregulation strategies. By addressing these disruptions, healthcare providers can better manage patients’ core temperatures, reduce the risk of hypothermia, and improve surgical outcomes, particularly in the vulnerable population of cancer patients.

Temperature monitoring

Accurate temperature monitoring during the perioperative period is crucial for ensuring effective thermoregulation and preventing hypothermia. Various techniques and sites can be used to measure body temperature, each with its advantages and limitations. The choice of temperature monitoring site and method depends on the clinical situation, the need for accuracy, and the invasiveness of the procedure. These sites and techniques along with their advantages and limitations are represented in Table 1 below.

**Table 1:** Comparative overview of various temperature monitoring techniques Abbreviations: PA: Pulmonary Artery, PAC: Pulmonary Artery Catheter, UC: Urinary Catheter.

Monitoring Site	Technique	Accuracy	Relevance	Limitations	Reference
PA	Placing a thermistor-tipped PAC	Highest	Ideal for critical patients and major surgeries where precise monitoring is crucial	Highly invasive, require specialized equipment, expertise personnel	[2,16]
Oesophageal	Insertion of a temperature probe into the oesophagus, usually at the lower third near the heart.	High	Commonly used in major surgeries, especially in intubated patients	Invasive (lesser than PA)	[2,17]
Bladder	Placement of a temperature probe via a UC into the bladder	High	Useful in prolonged surgeries and critical care	Lagging readings	[17]
Rectal	Insertion of a temperature probe into the rectum.	High, but lagged response	Alternative when other core measurements are not feasible	Invasive, uncomfortable	[17]
Nasopharyngeal	Placement of a temperature probe in the nasopharynx, close to the hypothalamus	High	Especially useful in head and neck surgeries	Placement challenging due to airway	[2,17]
Tympanic Membrane	Insertion of a thermocouple or infrared probe into the ear canal to measure the tympanic membrane temperature.	High, if properly placed	Non-invasive and quick, suitable for various settings	Requires correct placement for accuracy	[2,17]
Axillary and Oral	Axillary - Placement of a thermometer in the axilla (armpit). Oral - Placement of a thermometer under the tongue.	Moderate	Non-invasive, more suitable for non-critical monitoring	Less accurate, influenced by external factors	[17]
Skin	Use of adhesive temperature sensors on the skin surface.	Least for core body temperature	Useful for trend monitoring and less invasive scenarios	Less precision	[17]

In summary, core temperature monitoring sites such as the pulmonary artery, oesophagus, bladder, rectum, nasopharynx, and tympanic membrane provide higher accuracy and are more relevant in critical surgical settings. Peripheral sites like the axilla, oral cavity, and skin are less accurate but may be used for non-critical monitoring or in situations where less invasive methods are preferred. The selection of the appropriate site and method is essential for effective perioperative temperature management, especially in vulnerable populations such as cancer patients [2,15-18].

In resource-limited operating rooms, the major factors that influence the choice of temperature-monitoring techniques are cost and availability of equipment. Core monitoring methods, such as pulmonary artery or oesophageal probes, offer the greatest accuracy but are relatively expensive and require sophisticated anaesthesia setups. Bladder, nasopharyngeal, and axillary/oral thermometry are less expensive and widely available and thus represent practical alternatives for oncology surgery in resource-poor settings where the maintenance of normothermia is nonetheless critical [2,17,18].

Impact of hypothermia on cancer patients

Hypothermia significantly suppresses immune function, a critical concern for cancer patients who are often already immunocompromised. The body’s natural defence mechanisms, including white blood cell activity and cytokine production, are less effective at lower temperatures. This suppression can lead to a decreased ability to fight off infections, which is particularly dangerous for cancer patients undergoing surgery. Effective immune function is crucial for preventing postoperative infections and promoting overall recovery [19].

Cancer patients are at a heightened risk of infections due to their weakened immune systems. Hypothermia exacerbates this vulnerability by further impairing immune responses and disrupting normal physiological functions. SSIs are a common and serious complication associated with perioperative hypothermia. In a randomized controlled trial, the incidence of SSIs was significantly higher in hypothermic patients (19%) compared to normothermic patients (6%) [20]. These infections can lead to prolonged hospital stays, increased healthcare costs, and higher morbidity and mortality rates. On average, patients affected with perioperative hypothermia stay 2.6 days longer, which is approximately a 20% increase of hospitalization [20]. A meta-analysis suggested that maintaining normothermia can save approximately $2,500 per patient, primarily because of fewer infections, reduced blood transfusion requirements, and shorter hospital stays [21].

Ruetzler et al. (2018) show that hypothermia impairs systemic perfusion and drug handling; however, a direct link between hypothermia and worsened chemotherapy or radiotherapy outcomes has not been established yet [3]. These treatments already place significant stress on the body, and the additional burden of hypothermia can lead to increased toxicity and reduced efficacy. However, cooling devices, like “cooling gloves and socks” used “prophylactically during chemotherapy” have been shown to be effective as they “limit peripheral drug exposure” [3].

Hypothermia can cause significant cardiovascular strain, leading to complications such as arrhythmias, decreased heart rate, and reduced cardiac output. Cancer patients, who may already have compromised cardiovascular systems due to the disease or its treatments, are particularly vulnerable to these effects. Hypothermia-induced vasoconstriction can lead to increased blood pressure and decreased peripheral perfusion, further complicating the perioperative management of these patients. Maintaining normothermia is crucial for ensuring cardiovascular stability and preventing related complications [22].

Hypothermia can alter the pharmacokinetics and pharmacodynamics of medications used in cancer treatment, affecting their efficacy and safety. Lower body temperatures can slow down enzymatic processes in the liver and other organs responsible for drug metabolism, leading to prolonged drug action and increased risk of toxicity [23]. For example, anaesthetic agents, pain medications, and chemotherapeutic drugs may have altered effects in hypothermic conditions, necessitating careful monitoring and dose adjustments. Hypothermia can interfere with the body’s ability to metabolize and clear chemotherapeutic agents by slowing hepatic enzyme activity, which can increase toxicity and potentially reducing their effectiveness and leading to treatment resistance by slowing hepatic enzyme activity, which can increase toxicity and reduce therapeutic effectiveness [23]. Ensuring normothermia can help maintain the appropriate metabolism and clearance of these medications, optimizing their therapeutic effects and minimizing adverse reactions [2,23].

By understanding and addressing the impact of hypothermia on immune function, infection risk, treatment resistance, cardiovascular health, and medication metabolism, healthcare providers can improve perioperative care for cancer patients, leading to better outcomes and enhanced recovery.

Current perioperative thermoregulation practices

Effective perioperative thermoregulation involves a multi-phase approach that includes preoperative, intraoperative, and postoperative interventions. Each phase requires specific strategies to ensure that the patient’s core body temperature remains within a normal range, thus minimizing the risk of hypothermia and its associated complications. The current existing strategies for thermoregulation in different perioperative stages have been summarised in Table 2 below.

**Table 2:** Current methods for thermoregulation in different perioperative stages Abbreviations: CTM: Continuous Temperature Monitoring, FAW: Forced-Air Warming, CWD: Conductive Warming Devices.

Preoperative Phase	Intraoperative Phase	Postoperative Phase
Medical History	CTM	CTM
Physical Examination	FAW	Postoperative Warming (Blankets, Heated Mattresses Warm Fluids)
Baseline Temperature	CWD	Pharmacological Management
FAW	Intravenous Fluid Warming	
CWD	Humidification	

Preoperative phase techniques involve a strict patient assessment such as medical history evaluation, physical examination, and baseline temperature monitoring for thermoregulation. Medical history is essential to identify risk factors such as age, body mass index, existing comorbidities (e.g., diabetes, cardiovascular diseases), and medications that could influence thermoregulation. Thyroid dysfunction and adrenal insufficiency are significant in this context since both can severely compromise temperature thermal control since hypothyroidism reduces basal metabolic heat production. In the meantime, adrenal insufficiency results in the reduction of vasomotor tone and stress response, requiring awareness about perioperative hypothermia and medications that could influence thermoregulation [3,12]. Assessing the patient’s overall physical condition and identifying baseline body temperature as a reference point can be useful to identify perioperative temperature changes [2,12,24].

Pre-warming techniques such as forced-air warming (FAW) and conductive warming devices (CWD) are also used preoperatively for thermoregulation. Pre-warming the patient using FAW systems before anaesthesia induction has been shown to effectively reduce the incidence of intraoperative hypothermia. This involves using warming blankets or gowns that circulate warm air around the patient’s body. CWD such as conductive warming pads can also be used to pre-warm patients by providing direct heat to the skin, thereby increasing body temperature before surgery begins [24,25].

Intraoperative phase techniques include continuous temperature monitoring, FAW, CWD, and intravenous fluid warming, humidification, and fluid management for thermoregulation. Continuous monitoring of core temperature, using reliable methods such as oesophageal, nasopharyngeal, or tympanic membrane probes, allows for immediate detection of temperature deviations and timely interventions [17]. FAW systems maintain normothermia using generating warm air through a blanket or gown covering the patient, creating a warm microenvironment. The temperature and airflow of the warming system should be adjusted based on the patient’s needs and the duration of the surgery. CWD are often used in combination with FAW to provide comprehensive temperature management [2,24,25].

Warming intravenous fluids before administration helps prevent hypothermia caused by the infusion of cold fluids. Heating the fluids to body temperature before administering reduces the risk of a drop in core temperature [2]. Humidification of the gases used in anaesthesia can also prevent heat loss through the respiratory tract [26]. Intraoperative phase techniques include continuous temperature monitoring, postoperative warming techniques (warming blankets, heated mattresses, and warm fluids) and pharmacological interventions (tricyclic antidepressants) can increase core body temperature [2].

Among the available techniques, FAWS represent the most effective and widely accepted technique for preventing low body temperature during surgical procedures among general and cancer patients alike. FAWS provides steady, even heat that assists in maintaining stable core temperature even in long or complicated surgeries [2]. Pre-warming with FAWS, 20 to 30 minutes before anaesthesia, reduces the risk of intraoperative hypothermia in cancer patients, due to lower metabolic rate and less body fat due to cachexia. The passive methods of intraoperative warming usually include the administration of warmed intravenous fluids and the use of humidified gases, which are generally less effective when compared with active surface warming techniques [24]. Overall, FAWS, combined with pre-warming and warmed intravenous fluids, has the best effect on keeping patients' body temperature within the normal range during the entire perioperative period.

By implementing these comprehensive thermoregulation practices across all perioperative phases, healthcare providers can significantly reduce the risk of hypothermia and its associated complications, thereby improving patient outcomes.

Challenges in perioperative thermoregulation for cancer surgeries

Effective perioperative thermoregulation in cancer surgeries is challenged by a variety of patient-specific, procedural, and environmental factors. The key challenges involved in cancer surgery thermoregulation include the cancer stage and type, comorbidity status, chemotherapy and radiation therapy, age and frailty, psychological factors, surgical procedural complications, and environmental factors [2,9,10,15,17,19,22,23,27]. Understanding these challenges is crucial for developing targeted strategies to maintain normothermia and optimize patient outcomes.

Different types and stages of cancer can significantly influence the body’s ability to regulate temperature. Advanced cancers often result in higher metabolic demands, leading to increased heat production. Conversely, some cancers, particularly those affecting the endocrine system, can disrupt normal thermoregulatory processes. Additionally, impaired immune function, from the cancer itself or its treatments, can exacerbate the risk of perioperative hypothermia [19,22,28].

Coexisting medical conditions also impact the risk of hypothermia. Patients with cardiovascular conditions may have impaired peripheral circulation, reducing their ability to distribute and retain heat. This makes them more susceptible to hypothermia during surgery [27]. Complications of diabetes, such as peripheral neuropathy and autonomic dysfunction, impair thermoregulatory responses (sweating and shivering) thereby increasing the risk of temperature fluctuations [27]. Cancer patients often suffer from malnutrition and cachexia, leading to decreased body fat and muscle mass. This reduces the body’s insulation and heat production capabilities, making it harder to maintain normothermia [15,27].

Many chemotherapy agents cause chemotherapy-induced peripheral neuropathy (CIPN), which can alter thermal sensation and impair the body’s ability to detect and respond to temperature changes. Ami et al. (2017) identified that paclitaxel can induce hypothermia in mice, and thermoregulation provided more desirable outcomes compared to mice without thermoregulation [29]. As CIPN can cause peripheral neuronal damage, which can impact thermoregulation, one can assume that CIPN indirectly contributes to disrupted thermal regulation, however further studies on this topic are required [2,15,28].

Older adults experience age-related declines in thermoregulatory mechanisms, such as reduced vasoconstrictive and shivering responses. They may also have decreased subcutaneous fat, reducing their ability to retain heat. Frail patients have diminished physiological reserves and resilience to stress, including thermal stress. They are more vulnerable to perioperative hypothermia and its complications. Older and frail cancer patients require close monitoring and proactive warming strategies to prevent temperature-related complications [2].

Psychological distress, anxiety, and depression can activate the body’s stress response, increase metabolic rate and potentially impact thermoregulation. Patients experiencing significant psychological stress may have heightened thermogenic responses. Psychological disorders can disrupt the autonomic nervous system, impacting peripheral blood flow and heat distribution [2]. Altered sympathetic and parasympathetic tone may contribute to perioperative temperature instability [2,9,15]. 

Long surgeries increase the risk of cumulative heat loss. Prolonged exposure to the surgical environment and continuous impairment of thermoregulatory responses can result in significant temperature decreases. Extensive surgeries, such as radical resections, involve large areas of exposure, leading to greater heat loss. The more tissue exposed, the higher the risk of hypothermia due to convective and evaporative heat loss. Techniques such as laparoscopy and robotic-assisted surgeries, while minimally invasive, can still pose thermoregulatory challenges due to insufflation with cold gases and extended operating times [2,9,10,13].

Environmental factors also significantly influence hypothermia. Operating rooms are often kept at cooler temperatures to maintain a sterile environment and ensure the comfort of the surgical team. However, this can increase the risk of hypothermia for patients. Adjusting the ambient temperature and using warming devices can help mitigate this risk. High airflow rates and laminar airflow systems, designed to reduce infection risk, can increase convective heat loss. Balancing infection control with thermal comfort is essential for optimal patient outcomes. Proper patient positioning and draping are crucial for minimizing heat loss. Exposing large areas of the body or using inadequate insulation can increase the risk of hypothermia. FAW, CWD, and circulating water blankets are essential for maintaining normothermia. These devices should be appropriately placed and adjusted to ensure consistent warming throughout the procedure [2,13,17,24,25].

By addressing these challenges through comprehensive perioperative temperature management strategies, healthcare providers can improve thermoregulation in cancer surgeries, reduce the risk of hypothermia, and enhance overall patient outcomes.

Future research directions

Future research should focus on developing personalized thermoregulation protocols that consider individual patient factors such as age, body mass index, cancer type and stage, comorbidities, and treatment history. Personalized approaches can optimize warming strategies and improve outcomes. Conducting longitudinal studies to assess the long-term impact of perioperative hypothermia and the efficacy of thermoregulation interventions on cancer patient outcomes is crucial. These studies can provide valuable insights into how temperature management affects recovery, recurrence rates, and overall survival.

Comparative effectiveness research can help identify the most effective and cost-efficient thermoregulation technologies and practices. Comparing different warming devices, monitoring methods, and protocols in various surgical settings will guide evidence-based practice. Research should explore the integration of multiple warming techniques to enhance overall efficacy. For instance, combining FAW with conductive warming garments and pre-warmed intravenous fluids may provide superior results compared to single-modality approaches.

Optimizing the operating room environment, including temperature, humidity, and airflow, can provide practical guidelines for reducing heat loss during surgery. Studying the impact of workflow and operational factors on thermoregulation practices can identify areas for improvement, ensuring that temperature management interventions are effectively implemented. Exploring novel biomaterials for warming devices, such as phase-change materials that can store and release heat efficiently, could revolutionize perioperative thermoregulation. Investigating pharmacological agents that can enhance the body’s natural thermoregulatory mechanisms or mitigate the effects of hypothermia could provide additional tools for managing perioperative temperature.

The future of perioperative thermoregulation in cancer surgeries lies in the integration of advanced technologies, personalized approaches, and comprehensive research. By embracing these innovations and directions, healthcare providers can improve patient safety, enhance surgical outcomes, and reduce the incidence of hypothermia-related complications.

## Conclusions

Maintaining normothermia during cancer surgeries is a critical component of perioperative care. This review summarises the physiological mechanisms of body temperature regulation, the influence of hypothermia on cancer patients, and current strategies for the prevention of temperature-related complications. It also discussed issues exclusively related to cancer surgery and analysed different methods of warming in regard to supporting efficient temperature control. The clinical implication of this would be that active temperature management with the use of forced-air warming systems, pre-warming before anesthesia, and warmed intravenous fluids can prevent perioperative hypothermia, decrease postoperative complications, and achieve optimised surgical outcomes in cancer patients.
